# How to alleviate cardiac injury from electric shocks at the cellular level

**DOI:** 10.3389/fcvm.2022.1004024

**Published:** 2022-12-22

**Authors:** Pamela W. Sowa, Aleksander S. Kiełbik, Andrei G. Pakhomov, Emily Gudvangen, Uma Mangalanathan, Volker Adams, Olga N. Pakhomova

**Affiliations:** ^1^Frank Reidy Research Center for Bioelectrics, Old Dominion University, Norfolk, VA, United States; ^2^Laboratory of Molecular and Experimental Cardiology, Heart Center Dresden, Technische Universität Dresden, Dresden, Germany; ^3^Department of Cardiology and Angiology, University Hospital Tübingen, Eberhard Karls University of Tübingen, Tübingen, Germany; ^4^Department of Molecular and Cellular Biology, Faculty of Pharmacy, Wrocław Medical University, Wrocław, Poland

**Keywords:** defibrillation, Poloxamer 188, cardiomyocytes, membrane repair, apoptosis, microsecond pulsed electric field (μsPEF), nanosecond pulsed electric field (nsPEF)

## Abstract

Electric shocks, the only effective therapy for ventricular fibrillation, also electroporate cardiac cells and contribute to the high-mortality post-cardiac arrest syndrome. Copolymers such as Poloxamer 188 (P188) are known to preserve the membrane integrity and viability of electroporated cells, but their utility against cardiac injury from cardiopulmonary resuscitation (CPR) remains to be established. We studied the time course of cell killing, mechanisms of cell death, and protection with P188 in AC16 human cardiomyocytes exposed to micro- or nanosecond pulsed electric field (μsPEF and nsPEF) shocks. A 3D printer was customized with an electrode holder to precisely position electrodes orthogonal to a cell monolayer in a nanofiber multiwell plate. Trains of nsPEF shocks (200, 300-ns pulses at 1.74 kV) or μsPEF shocks (20, 100-μs pulses at 300 V) produced a non-uniform electric field enabling efficient measurements of the lethal effect in a wide range of the electric field strength. Cell viability and caspase 3/7 expression were measured by fluorescent microscopy 2–24 h after the treatment. nsPEF shocks caused little or no caspase 3/7 activation; most of the lethally injured cells were permeable to propidium dye already at 2 h after the exposure. In contrast, μsPEF shocks caused strong activation of caspase 3/7 at 2 h and the number of dead cells grew up to 24 h, indicating the prevalence of the apoptotic death pathway. P188 at 0.2–1% reduced cell death, suggesting its potential utility *in vivo* to alleviate electric injury from defibrillation.

## Highlights

- AC16 human cardiomyocytes subjected to μsPEF shocks display strong activation of caspase 3/7, and the number of dead cells gradually increases up to 24 h, indicating the prevalence of apoptotic death.
- After exposure to nsPEF shocks most of the lethally injured cardiomyocytes die within 2 h, with little or no caspase 3/7 activation.
- Poloxamer 188 profoundly reduces cardiomyocytes death after ns- and μsPEF shocks, highlighting its potential use to enhance cardiomyocyte recovery after defibrillation.

## 1 Introduction

Each year, approximately 350,000 adults in the United States suffer out-of-hospital cardiac arrests (OHCA). In 19%, the initial recorded cardiac rhythm is shockable by defibrillation (1). However, the survival statistics for OHCA remain disappointing. According to the American Heart Association, the survival to hospital discharge after an incidence with shockable rhythm reaches 25% (2). The high mortality from cardiac arrest can in part be contributed to the electrical injury from defibrillation, compromising myocardial function (3–8). Indeed, many adverse outcomes of defibrillation are associated with electroporation (9–12), including subepicardial tissue damage (11, 12) and proarrhythmic activity (13). At the same time, electroporation, transiently impairing the conduction, can be antiarrhythmic and support defibrillation (9, 14).

In comparison to conventional millisecond shocks used by defibrillation, much shorter nanosecond shocks require higher field strength but overall lower energy (15). “Nanoelectropores” in the cell membrane after nsPEF shocks are 1–1.5 nm in diameter (16, 17) and tend to be smaller than those formed by millisecond shocks (18–20), thus leakage through the membrane is less injurious than after conventional defibrillation. In mouse, pig, and rabbit cardiomyocytes, nanosecond shocks resulted in less uptake of membrane impermeable propidium dye compared to longer shocks thus indicating reduced cell injury (21). They are also expected to induce spatially uniform electroporation which implies more homogenous tissue penetration (20, 22, 23). nsPEF shocks were found to cause calcium entry into cardiac myocytes by routes other than calcium channels, followed by a slow sustained depolarization of unknown significance to cardiomyocytes (21, 24).

The efficiency and safety of ns defibrillation in the termination of fibrillation have already been demonstrated in isolated rabbit hearts (15). Interestingly, *in vitro* stimulation of single cardiomyocytes was impossible without electric damage (21, 24). The potential explanation is that stimulation of the whole heart results from electroporation of single cells, which further stimulate the adjacent ones. A single stimulation of the entire heart would therefore cause undetectable lesions (25). Whether the excitation by nsPEF shocks is possible without electroporation and which mechanism prevails is determined by the membrane charging time constant τ (26). Since this parameter is proportional to cell size, cardiomyocytes, which are among the largest cells, are likely to have a higher time constant. Moreover, in the heart tissue, due to the lower extracellular conductivity and gap junctions between neighboring cells, the charging time constant may be substantially increased. Increasing τ shifts the balance from electroporation towards excitation, The larger time constant *in situ* may be a feature that enabled defibrillation of the heart without damage (26).

Electroporated cells could still be capable of restoring their membrane continuity, thus possibly avoiding cell death (25, 27–31). During CPR, the delay needed for membrane restoration can be fatal. This prompted our study to search for an agent that could mitigate electroporation-induced membrane damage of cardiomyocytes. One of the promising substances for this task is Poloxamer P188 (P188), a member of the copolymer group. P188 was shown to stabilize cellular membranes (32) and protect against electrical injury, increasing the survival of electroporated isolated rat skeletal muscle cells (33) and restoring the structural integrity of muscle tissue (34). Regarding cardiac injury during CPR, the intervention with Poloxamer 188 has been evaluated only as part of a bundle therapy with standard advanced life support (35). Therefore, its role in preventing cardiac electric damage has not been established.

Here, we hypothesized that P188 would alleviate electric insult and reduce cellular death of human cardiomyocytes when infused straight after nanosecond or microsecond electric shocks. Using a human cardiomyocyte cell line (AC16), we observed a strong protective effect of P188 after ns- and μsPEF shocks. We established different dynamics and mechanisms of cell killing by ns- and μsPEF shocks. Our data suggest that exposure to nanosecond pulses induced necrosis and microsecond pulses likely activated apoptosis of human cardiomyocytes. Our research thus implies the protective effect of P188 against both types of cell death.

## 2 Materials and methods

### 2.1 Cell culture

AC16, a human cardiomyocyte cell line derived from adult ventricular heart tissues (36) was purchased from Millipore (Burlington, MA, USA). Cells were maintained at 37°C, 5% CO_2_ in DMEM/F-12 medium (Gibco, New York, NY, USA) containing 2 mM L-Glutamine (Mediatech Cellgro, Herndon, VA, USA), 12.5% FBS (Atlanta Biologicals, Norcross, GA, USA) and 1X Penicillin-Streptomycin Solution (GIBCO, Gaithersburg, MD, USA), and expanded to the final confluency of 80–90%. Complete medium formulation is available at: https://www.thermofisher.com/de/de/home/technical-resources/media-formulation.55.html.

### 2.2 Electroporation of cell monolayers

We used nanofiber 24-multiwell plates (Nanofiber Solutions 9602, Darmstadt, Germany) to create monolayers of AC16 human cardiomyocytes. The plates were precoated with 20-μm thick layers of nanofiber polymers of 700-nm diameter, which structurally mimic the architecture of the cardiac extracellular matrix (37). We also coated the plates with human fibronectin (Gibco, Carlsbad, CA, USA) dissolved in PBS (Gibco, Carlsbad, CA, USA) at 2 μg/cm^2^ to minimize cell detachment. One day before exposure cells were seeded on the plates at 0.15 × 10^6^ cells per well to achieve a homogenous monolayer.

Anet A8 3D printer (Shenzhen Anet Technology Co, China) was customized with an electrode holder to precisely position stimulation electrodes orthogonal to cell monolayers (38). Contact electrodes produced the electric field gradually decaying with distance from them, allowing the comparison of cell killing in a range of electric field strengths in a single sample. A 24-well plate was precisely fixed in a frame attached to the printer bed. The printer was programmed to deliver the electrodes to the center of the first well, remain there for 40 s for PEF exposure, then move to the second well, and so on. This interval was set to 40 s to maintain a constant interval between PEF treatments, agent administration, and image acquisition. Since the maximal dose of P188 applied *in vitro* in earlier studies was about 1% (34, 39, 40), we tested three different concentrations of P188: 0.2% (0.2 mM), 0.5% (0.6 mM) and 1% (1.2 mM).

The compound was diluted in the medium in a double concentration and the volume of added solution was equal to the volume of medium already in a well. The agent was administrated manually using pipette 10 s after PEF exposure. The same volume of medium without P188 was added as a vehicle control.

**FIGURE 1:**
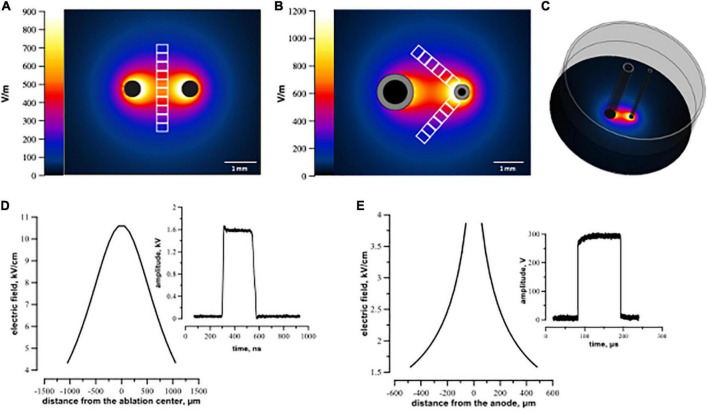
Electric field simulation for symmetric **(A,D)** and asymmetric **(B,C,E)** electrode arrays. Panels **(A,B)** visualize the calculated electric field distribution with 1 V applied between the electrodes. The direction in which the electric field thresholds were measured is indicated by white rectangles representing regions of interest (see text). Panel **(C)** illustrates a 3D configuration of the asymmetric electrode assembly orthogonal to the plastic well. Panels **(D,E)** show the electric field values for 300 V (100 μs pulse) and 1.74 kV (300 ns pulse) applied between electrodes. Distance from the ablation center **(D,E)** was measured along the regions of interest from the center of ablation (the middle of the line connecting centers of the electrodes) for symmetrical electrodes **(D)** and from the middle of the anode for the asymmetrical electrode **(E)**.

In the experiments, we used two different electrode arrays, an asymmetric one for μsPEF shocks and a symmetrical one for nsPEF shocks. Previous studies with trains of long pulses (≥100 μs) at lethal intensities reported intense bubble formation at the cathode (21, 38). Bubbles covering the electrode surface could change the electric field distribution during a pulse train. To avoid this effect, we made the cathode electrode larger, thereby decreasing the current density at its surface (Figure 1). Lower current density reduces gas evolution at the electrode (41). Using a larger electrode, we did not observe bubble formation.

The asymmetric electrodes used for μsPEF shocks were made of stainless steel hollow rods, 1.2 mm outer diameter for cathode and 0.5 mm for anode. They were placed 2.2 mm apart (center-to-center), parallel to each other and orthogonal to the cell monolayer.

Electroporation with nsPEF shocks was performed by applying electric pulses between two symmetrical cylindrical electrodes made of tungsten (500 μm diameter, 1.8 mm center-to-center distance). In preliminary experiments, electric field parameters for micro- and nanosecond pulses (number of pulses, frequency) were optimized to create comparable lesions. Trains of 200, 300-ns pulses at 1.74 kV were delivered at 10 Hz frequency from an EPULSUS-FPM4-7 pulse generator (Energy Pulse Systems, Lisbon, Portugal). Trains of 20, 100-μs pulses at 300 V were delivered at 1 Hz from a 6040 Universal Pulse Generator (BNC, San Rafael CA) triggered by a Model 577 Digital Delay/Pulse Generator (BNC). Pulse shape and amplitude were continuously monitored with a TDS 3052 oscilloscope (Tektronix, Beaverton, OR, USA).

### 2.3 Experiment protocol

For fluorescence imaging, 75 μM propidium iodide (PI) (Thermo Fisher Scientific, Waltham, MA, USA), an established membrane permeability marker dye, and 2.25 μM Hoechst-33342 (Thermo Fisher Scientific, Waltham, MA, USA), a membrane-permeant nucleic acid stain, were added 20 min prior to imaging, starting from 2 h after ns- and μsPEF shock treatments. PI is membrane impermeant and has weak fluorescence in the solution. However, once the cell membrane is permeabilized, Propidium enters the cell and its emission increases by binding to intracellular nucleic acids (21). Given that most membrane repairs are done within 10–15 min after exposure (31, 42–44), we considered that adding PI at 2 h after electroporation or later labeled only permanently permeabilized (i.e., dead) cells. All PI-positive cells were also positive for Hoechst, which made them look pink when both fluorescence channels were combined. PEF shocks were delivered in the culture medium. Each time point after PEF exposure was measured in a separate well to exclude possible cytotoxicity effect of Hoechst dye. We limited pulse treatments to 8 wells on each plate, so the plate remained outside the incubator no longer than 6 min.

### 2.4 Caspase 3/7 activity detection

For the detection of caspase activity at 1, 2, and 4h after exposure to PEF, the medium was replaced with 6 μM of Caspase 3/7 Green Detection Reagent CellEvent (Invitrogen) dissolved in 200 μl PBS with 5% FBS, mixed with PI and Hoechst and returned to the incubator for another 30 min before imaging. As a positive control, cells were incubated with 0.5 μM apoptotic inducer staurosporine (Sigma-Aldrich St. Louis, MO, USA) for 6 h.

### 2.5 Image acquisition

**FIGURE 2:**
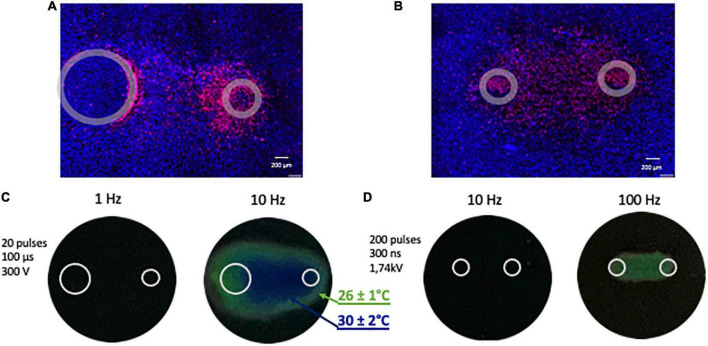
Representative images illustrating fluorescent staining of human cardiomyocyte monolayers and temperature monitoring. **(A)** Cells were subjected to 20 pulses (100 μs duration, 1 Hz, 300 V) and **(B)** 200 pulses (300 ns duration, 10 Hz, 1.74 kV). Panels **(A,B)** show merged signals from Hoechst-33342 (blue) and propidium iodide (red). Gray circles mark the footprints of the asymmetrical **(A)** and symmetrical **(B)** electrodes. **(C,D)** Monitoring the temperature rise following PEF (pulsed electric field) exposure with thermochromic liquid crystal sheets. Images were taken at the end of the exposure. Temperatures at the color transitions are labeled in the legend.

We used wide-field fluorescence microscopy for measuring cell death caused by nano- and microsecond electric shocks (Figures 2A, B). Images were acquired using an IX83 microscope (Olympus America, Hamden, CT, USA) custom configured with an automated MS-2000 scanning stage (ASI, Eugene, OR, USA), X-Cite 110LED illuminator (Excelitas Technologies Corporation, Waltham, MA, USA), and an ORCA-Flash4 sCMOS camera (Hamamatsu, Shizuoka, Japan). A total of 9 images of adjacent regions (a 3 × 3 square) were taken using a 10x, 0.38 NA objective and merged into a single high-resolution image with cellSens software (Olympus America, Hamden, CT, USA). The samples were imaged using DAPI, Cy3 and FITC filter sets for Hoechst, PI, and caspase 3/7 signals, respectively.

### 2.6 Electric field simulations

Electric field (E) distribution maps were generated by a numerical simulation with Sim4Life v3.2 software (Zurich Med Tech, Swiss). The electrodes were placed in the same position as in experiments described in Section “2.2 Electroporation of cell monolayers.” Separate simulations were performed for symmetrical (Figures 1A, D) and asymmetrical (Figures1B, C, E) electrode assemblies. The electric field values in Figure 1 were calculated in a plane 5 μm above the bottom of the well for 1 V applied between the electrodes. For further data analyses, the simulated electric field values were multiplied by the applied voltage, i.e., by 300 V for μs- and 1.74 kV for nsPEF shocks.

### 2.7 Image analysis

For cell death analysis after nsPEF shocks, we drew 10 rectangular 600 μm × 180 μm regions of interest (ROI) in the middle of the line connecting the centers of two symmetrical electrodes footprints (Figure 1A). Since we used asymmetrical electrodes for μsPEF shocks, we set two lines arising from the anode at an angle of 45° to the line connecting the centers of electrodes and designated along them 12 rectangular 600 μm × 180 μm ROIs (Figure 1B). In each ROI, cells stained with PI and cells stained with Hoechst only were counted with cellSens software. The percentage of dead cells was calculated as a ratio of PI-stained cells to all cells. For data processing, we matched the values of cell death from each ROI with the corresponding electric field intensity obtained from simulations (see Section “2.6 Electric field simulations”). The cell death ratio leveled off to a plateau (about 5%) away from the electrodes, indicating that in this area the effect of electric field was negligible. Note that the cells located in this peripheral region were subjected to the same conditions. Consequently, ROI covering those areas were regarded as sham control.

For Caspase detection we applied the same principle, however, for asymmetrical electrodes ROIs (600 μm × 180 μm) were drawn in the middle between electrodes, since in the nearest area of electrodes the majority of cells were PI-permeable before caspase activity could be detected. In each ROI, using cellSens software, we calculated the integrated fluorescence intensity (the sum of each pixel intensities).

### 2.8 Thermometry

To rule out the thermal effect during exposure to electric shocks, maximum heating from PEF treatments was assessed (38, 45; Figures 2C, D). R25C5B calibrated thermochromic liquid crystal sheets (LCR Hallcrest, Glenview, IL, USA) were positioned at the bottom of the well in contact with electrodes, and color changes were registered with a digital camera. All measurements were taken at a room temperature of 22–23°C. No color changes were observed when a train of 200, 300 ns pulses at 10 Hz or 20, 100 us pulses at 1 Hz was applied, which are the repetition rates used in our experiments. For positive control, we increased the frequency of ns- and μsPEF shocks to 100 and 10 Hz, respectively, and the color changed from black, implying no detectable heating effect, to green (26 ± 1°C) and blue (30 ± 2°C).

### 2.9 Statistical analysis

In all experiments, different exposure conditions (i.e., P188 concentration) were randomized. Exposure of one well was considered as a single experiment. Each graph represents the results of 3 to more than 6 experiments. Data were analyzed using a two-tailed *t*-test with Holm-Sidak correction for multiple comparisons. Normal distribution of data was confirmed using D’Agostino and Pearson normality test. Grapher 11 software (Golden Software, Golden, CO, USA) was used to prepare the graphs.

## 3 Results

### 3.1 The time course of cell death following ns- and μsPEF shocks

Cell death caused by pulsed electric fields can occur over extended time intervals (46, 47). Our first experiments were designed to determine the time interval after PEF treatment when the cell death reaches maximum. A train of 200, 300-ns pulses at 10 Hz, or of 20, 100-μs pulses at 1 Hz was applied to cell monolayers.

**FIGURE 3:**
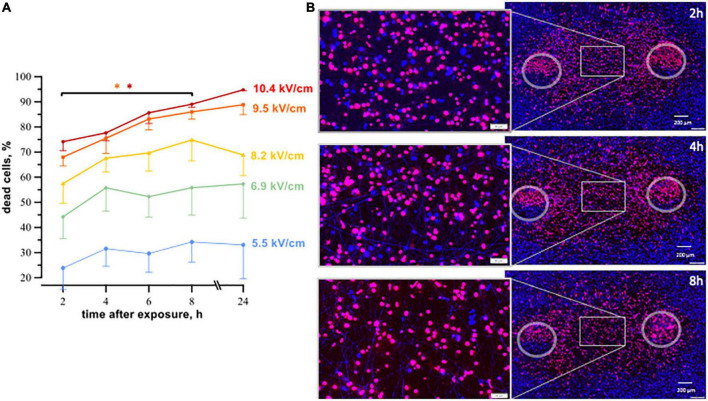
The time course of cell death in human cardiomyocytes subjected to nanosecond electric shocks (200 pulses at 10 Hz, 300 ns, 1.74 kV). **(A)** The percentage of dead cells in the regions with different electric field strength as a function of time after nsPEF shocks exposure, mean ± SEM, 5–6 independent experiments for each time interval, *p < 0.05 for the difference in the percent of dead cells between different time intervals. Cells stained with propidium (red dye) were considered dead, while Hoechst staining (blue) marked live cells. Panel **(B)** represents fluorescence images of human cardiomyocytes following nsPEF shocks applied to the cell monolayer. Gray circles mark the footprints of the symmetrical electrodes. Insets show magnifications of selected areas. The cell death reached its maximum at 8 h after exposure.

After nsPEF shocks (Figure 3) the percentage of dead cells was gradually increasing up to 8 h. For example, at 9.5 kV/cm, the percentage of dead cells rose from 68% ± 3 at 2 h to 86% ± 3 at 8 h, *p* < 0.05. At longer intervals, up to 24 h this percentage did not vary. Most of cell death occurred already at 2–4 h, and the further (later) increase in cell death was minor.

**FIGURE 4:**
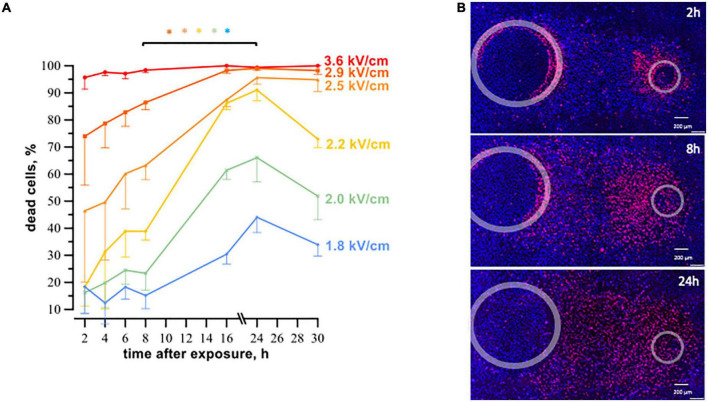
The time course of cell death in human cardiomyocyte subjected to microsecond electric shocks (20 pulses at 1 Hz, 100 μs, 300 V). **(A)** The plots display the percentage of dead cells in the regions with different electric field strength as a function of time after μsPEF shocks exposure, mean ± SEM, 3–5 independent experiments for each time interval, *p < 0.05 for the difference in the percent of dead cells between different time intervals. Cells were labeled with propidium and Hoechst at time intervals ranging from 2 to 30 h after μsPEF shocks see Figure 3 and text for more details. Panel **(B)** represents fluorescence images of human cardiomyocytes following μsPEF shocks. Gray circles mark the footprints of the asymmetrical electrodes. Note the gradual increase of cell death over time with a maximum at 24 h.

Cell death after exposure to μsPEF shocks peaked after an even longer interval of time (Figure 4). For most electric field strengths, cardiomyocyte death was gradually increasing to its maximum at 16–24 h. The only exception was with the strongest tested electric field of 3.6 kV/cm, which killed nearly 100% of cells already at 2 h. The highest (5-fold) increase in cell death with time was observed at 2.2 kV/cm, where the percentage of dead cells rose from 18% ± 7 at 2 h to 91% ± 4 at 24 h (*p* < 0.05). The apparent decline in cell death from 24 to 30 h after μsPEF shocks can be explained by the repopulation of the ablated area by viable cells and/or by the detachment of dead cells.

Considering that cardiomyocyte death in the experiments above did not occur immediately after PEF exposure, in the following experiments, cell death was measured 4- and 8 h after nsPEF shocks and 8- and 24 h after μsPEF shocks exposure.

### 3.2 P188 profoundly reduces cardiac cell death after nsPEF shocks and μsPEF shocks

**FIGURE 5:**
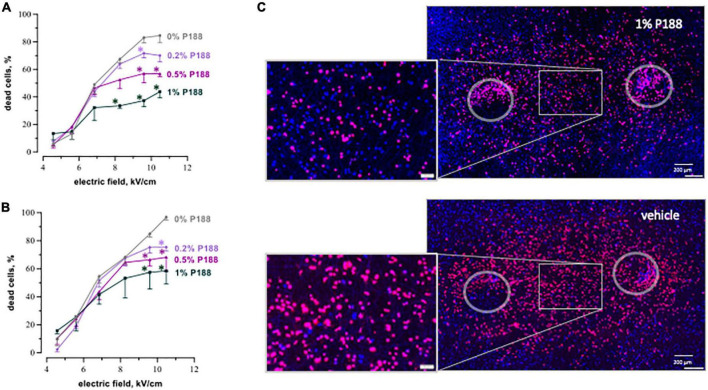
P188 reduces cardiac cell death after nsPEF shocks. The plots display the percent of dead cells at 4 h **(A)** and 8 h **(B)** after exposure to 200, 300-nsPEF shocks at 10 Hz. Poloxamer 188 (P188) was added at indicated concentrations (0% is vehicle control) 10 s after the end of nsPEF shocks exposure. Mean ± SEM, 3–5 independent experiments for each P188 concentration. *p < 0.05 for the difference between vehicle and P188 at the same electric field strength. Fluorescence images of cell monolayers **(C)** were taken at 8 h after exposure to nsPEF shocks followed in 10 s by either 1% P188 or vehicle. Selected areas are magnified in the insets. Note a profound reduction of cell death in samples treated with P188. See Figure 3 for more details.

P188 applied 10 s after nsPEF shocks decreased cell mortality in all tested concentrations, with more protective effect for stronger (more lethal) electric field exposures. Figure 5 presents cell death measurements at 4 and 8 h after nsPEF shocks exposures at different electric field strengths. The protective effect of P188 was both concentration- and electric field-dependent, becoming stronger and statistically significant for exposures to the electric field of 8.2 kV/cm or higher. At 8 h after exposure at 10.4 kV/cm, 1% P188 reduced cell death 1.7 times compared to the vehicle control (from 97% ± 2 to 58% ± 9, *p* < 0.05). Treatments with 0.5 and 0.2% P188 reduced cell killing 1.4 times (from 97% ± 2 to 68% ± 10) and 1.3 times (from 97% ± 2 to 75% ± 3), respectively (*p* < 0.05).

P188 at all concentrations reduced or eliminated the increase of cell killing as the electric field was made stronger, resulting in a plateau in cell death curves (Figures 5A, B). This result could indicate that the additional cell death at these stronger electric fields was due to a different mechanism than with weaker electric fields, and that the cell death mechanism triggered by stronger fields was selectively sensitive to P188. A more in-depth analysis of this phenomenon was beyond the scope of this study but is considered for future work.

**FIGURE 6:**
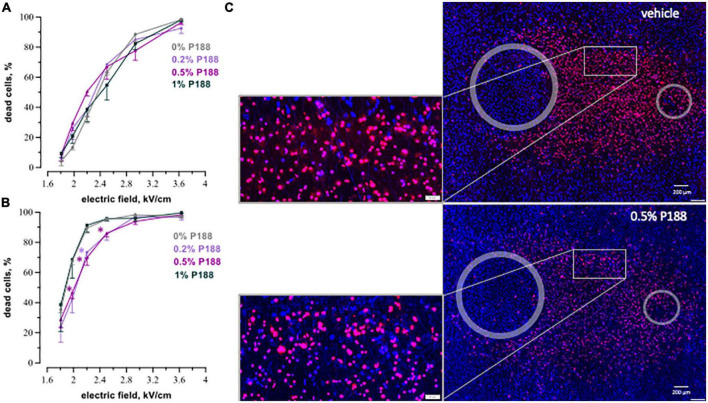
P188 protects cardiac cells against microsecond electric pulses. The plots display the percent of dead cells at 8 h **(A)** and 24 h **(B)** after exposure to 20, 100-μsPEF shocks at 1 Hz. P188 was added at indicated concentrations (0% is vehicle control) 10 s after the end of μsPEF exposure. Mean ± SEM, 3 experiments for each P188 concentration. *p < 0.05 for the difference between vehicle and P188 at the same electric field strength. The protective effect became significant at 24 h for P188 at 0.5% at the electric field strengths from 2.0 to 2.5 kV/cm and at 0.2% at 2.2 kV/cm. Panel **(C)** displays the example fluorescence images of cell monolayers at 24 h after exposure to μsPEF shocks.

P188 protected cardiomyocytes against μsPEF shocks injuries as well (Figure 6). The protective effect became significant only at 24 h (Figure 6B). P188 at 0.2 and 0.5% reduced cell death 1.2–1.6 times (*p* < 0.05) at the electric field strengths from 2.0 to 2.5 kV/cm. There was no protective effect at either lower or higher field strengths, probably because of just modest lethality and damage too severe to repair, respectively. Unlike the data for 8 h after nsPEF shocks exposure, 1% P188 did not reduce cell death at 24 h after μsPEF shocks. The protection could be counterbalanced by cytotoxicity of P188 at this highest concentration (39).

Promising outcomes of P188 treatment together with the different timing of cell death after μs- and nsPEF shocks motivated further study to investigate the cell death mechanisms. A vast fraction of cardiomyocytes treated with nsPEF shocks died at intervals shorter than required to accomplish the apoptotic death pathway (48), whereas a 16–24 h delay to cell death after μsPEF shocks suggested apoptosis.

### 3.3 100 μsPEF shocks cause apoptosis in human cardiomyocytes

**FIGURE 7:**
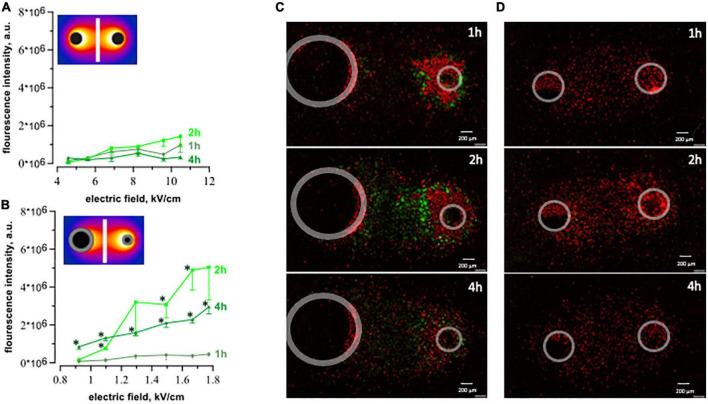
Caspase 3/7 activation after exposure to 20 pulses with 100 μs duration (1 Hz, 300 V) and 200 pulses with 300 ns duration (10 Hz, 1.74 kV). The plots display integrated fluorescence intensity of caspase 3/7 activity calculated for each region of interest (see text for details). Fluorescence was measured at 1-, 2-, and 4 h after exposure to ns- **(A)** and μsPEF shocks **(B)**. Every single plot represents 3–5 independent experiments, *p < 0.05 for the difference between 1 h and 2- or 4 h. We observed an increase in caspase activity (green dye) following μsPEF shocks **(C)**, but not after nsPEF shocks **(D)**.

We measured caspase 3/7 activity for the detection of apoptosis. Results confirmed the apoptotic death pathway after μsPEF shocks (Figure 7). Cells exposed to 20, 100 μs pulses at 1 Hz displayed a significant increase in caspase 3/7 activity at 2 and 4 h compared to 1 h after pulse delivery (*p* < 0.05), with the highest fluorescence intensity at 2 h. This time interval of caspase detection corresponds to the early stage of apoptosis (49, 50). Caspase 3/7 activation following nsPEF shocks was marginal (Figures 2A, D). A large number of cells became PI-permeable before caspase activity could be detected.

## 4 Discussion

The novel methodology of our experiments enabled labor-effective, concurrent measurements of cardiac cell death for a range of the electric field strength in a single experiment. A customized 3D printer with mounted electrodes delivered electric pulses directly to the cell monolayer bypassing the process of cells trypsinization and repetitive pipetting, which might affect cell physiology (51, 52). This approach excluded the influence of enzymatic proteolysis and shear stress, providing more reliable results.

P188 has been shown to promote membrane repair in various cells and tissues (32, 34, 39, 53–57). The work of Lee et al. (34) was of particular interest to us, showing that P188 effectively prevents skeletal muscle membrane disruption caused by electroporation when used in concentrations > 0.05%. P188 was also shown to restore structural integrity of electropermeabilized muscle tissue *in vivo*, yielding a peak blood level of 0.8% (34). Conversely, studies by Quinlan et al. (58) found no effect of P188 in preventing exercise-induced cell membrane breakdown in dystrophin-deficient mdx skeletal muscle fibers. However, the authors suggested that the lack of effect might be attributed to the low concentration of P188 used in the study. Serum levels measured immediately before and during the experiment averaged 0.15% P188.

Our study produced several major findings. We demonstrated a different time course of ns- and μsPEF shocks -induced cardiomyocyte death. The majority of cells exposed to nanosecond shocks was dead within the first 2 h even at the lowest field strength. Whereas after microsecond shocks only a small fraction of cells subjected to the highest electric field died within 2 h after exposure. When exposed to nsPEF shocks, cell death reached its maximum at 8 h. After μsPEF shocks cell death was gradually increasing to its peak at 16–24 h. It is commonly known that electric pulses can impose a different mechanism of cell death depending on the cell type (59, 60). Here, we presented that AC16 human cardiomyocytes exposed to pulses of different duration can undergo either apoptotic or necrotic cell death. Cell death mechanism after electroporation of different cardiomyocytes cell lines remains an area for future investigation.

Several studies support our finding that 100 μs pulses are capable of triggering the apoptotic death pathway (61–64). Nevertheless, the majority of reports associate nsPEF shocks with apoptosis or other programmed forms of cell death (22, 46, 47, 59, 60, 65–68) showing caspase activation (66), cytochrome C release into the cytoplasm (65), externalization of phosphatidylserine (PS) (66), poly ADP-ribose polymerase (PARP) cleavage (46), and inter-nucleosomal DNA fragmentation (66). However, further studies found that the nsPEF shocks -induced externalization of PS was associated with electroporative membrane damages rather than activation of the programmed death pathway (17, 69). At the same time, nsPEF shocks -treated cells have been observed to swell right after exposure (18, 47, 70, 71), which is a morphological hallmark of necrosis. The prevailing cell death mechanism in cells exposed to PEF may be influenced by ionic balance, ATP supply, and the level of intracellular damage (46). In addition, the increase in the number of pulses or their amplitude and frequency can determine the cell death mechanism (60, 72).

The interplay between the induction of apoptosis and necrosis is not simple and depends not only on pulse parameters but, for example, on the presence of Ca^2+^ in the medium and on the colloid-osmotic composition of the medium (47, 73). That said, numerous studies reported that it is the distinguishing feature of nsPEF shocks that they induce apoptosis, as opposed to necrotic cell death from longer pulses (65, 66, 68). Our study is the first report that for a comparable membrane cell killing efficiency (tested in the wide range of the electric field strengths) the pathway to cell death is the opposite, namely necrosis with nsPEF shocks and apoptosis with μsPEF shocks. Comparing our data with other studies, we suspect that it is unlikely just the train duration, pulse frequency or peak electric field (which do not differ much from other studies) but the unique physiology of the cell line we studied and which makes it react differently. This unique observation may bring the important keys to understanding what are the physiological features responsible for one or another cell death pathway, and they will certainly be our focus for future work.

Furthermore, we demonstrated that the application of 0.2-, 0.5-, and 1% P188 significantly reduced the death of cardiomyocytes exposed to nsPEF shocks. Similar protection against microsecond PEF shocks was demonstrated with 0.5 and 0.2% P188.

According to several earlier reports, P188 can prevent necrosis (53, 56, 74) as well as apoptosis (75, 76). In our study P188 efficiently suppressed cardiomyocyte death imposed by nsPEF shocks. P188 was also efficient against μsPEF shocks, capable to activate caspase 3/7 and thus suggesting protection against apoptosis, although it showed no effect in higher 1% concentration. Presumably, this higher concentration after a long 24-h incubation became toxic in apoptotic cells. Based on our study, it appears appropriate to conduct subsequent *in vivo* measurements to test the efficacy of this agent for longer observation time.

P188 is the only copolymer molecule approved by the U.S. Food and Drug Administration as safe for oral or intravenous administration (77, 78) when dosed for up to 30 mg/kg/h for 72 h to reduce blood viscosity before transfusion (79). The expanding list of patents for P188 suggests that it may also be useful in preventing cell membrane damage in a variety of medical conditions. The authors of the patent *Methods and Compositions of a Polymer (Poloxamer) for Repair of Electrical Injury* suggested its possible application for victims of electrical trauma (80). In cardiology, it is expected to prevent cardiomyopathy (81) and chronic progressive heart failure (82). Our study highlighted the potential use of P188 to enhance cardiomyocyte recovery after defibrillation.

The beneficial effect of P188 in preventing cardiomyocyte electric injury can be attributed to its structure. Hydrophilic ends of P188 adsorb to membrane bilayers and interact with hydrocarbon tails of lipids. This is thought to reduce the size and number of electropores (83). P188 raises the electroporation threshold (84) and facilitates resealing of the cell membrane (34). The action of poloxamers may be selective to damaged portions of electroporated membranes. Namely, P188 would only interact with compromised bilayers, increasing locally reduced lipid packing density (85). Once the membrane is reconstituted, higher surface pressure will likely displace Poloxamer from the membrane (85).

Cardiac damage after defibrillation raises concerns about its contribution to the poor outcome of CPR. Sarcolemma injuries have been found after ms and ns electric shocks (12, 24). Since both types of pulses damage the membrane, reinforcement of membrane restoration might be necessary to prevent cell death. The clear benefits of P188 application after nanosecond pulses warrant further evaluation of ns-defibrillation with the perspective of improvement in post-cardiac arrest mortality.

An alternative solution would be to reduce the harm of electrical pulses in general. One of the promising modalities of nanosecond shocks for defibrillation without cardiac damage is MHz compression of nsPEF shocks. Applying high-rate bursts enabled cardiomyocytes excitation at lower voltage than with a single ns shock, giving a large safety gap (86).

## Data availability statement

The raw data supporting the conclusions of this article will be made available by the authors, without undue reservation.

## Author contributions

PS, OP, and AP conceived the study and analyzed and interpreted the data. AK and PS performed experiments. EG created software. PS performed electric field simulations and created figures. OP supervised the study. PS wrote the manuscript, with contributions and editing by all authors. All authors contributed to the article and approved the submitted version.
