# Supplementary figures and images for: How to alleviate cardiac injury from electric shocks at the cellular level
Source: Front Cardiovasc Med. 2022 Dec 22;9:1004024. doi: 10.3389/fcvm.2022.1004024 (PMC9812960; doi:10.3389/fcvm.2022.1004024)

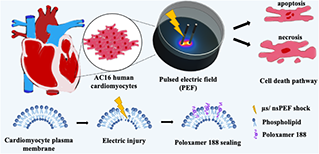

Supplement: Supplementary file 1 [file Image_1.TIFF]
